# Toughening Fe-based Amorphous Coatings by Reinforcement of Amorphous Carbon

**DOI:** 10.1038/s41598-017-04504-z

**Published:** 2017-06-22

**Authors:** Wei Wang, Cheng Zhang, Zhi-Wei Zhang, Yi-Cheng Li, Muhammad Yasir, Hai-Tao Wang, Lin Liu

**Affiliations:** School of Materials Science and Engineering and State Key Laboratory of Material Processing and Die & Mould Technology, Huazhong University of Science and Technology, Wuhan, 430074 China

## Abstract

Toughening of Fe-based amorphous coatings meanwhile maintaining a good corrosion resistance remains challenging. This work reports a novel approach to improve the toughness of a FeCrMoCBY amorphous coating through *in-situ* formation of amorphous carbon reinforcement without reducing the corrosion resistance. The Fe-based composite coating was prepared by high velocity oxy-fuel (HVOF) thermal spraying using a pre-mixed Fe-based amorphous/nylon-11 polymer feedstock powders. The nylon-11 powders were *in-situ* carbonized to amorphous carbon phase during thermal spraying process, which homogeneously distributed in the amorphous matrix leading to significant enhancement of toughness of the coating. The mechanical properties, including hardness, impact resistance, bending and fatigue strength, were extensively studied by using a series of mechanical testing techniques. The results revealed that the composite coating reinforced by amorphous carbon phase exhibited enhanced impact resistance and nearly twice-higher fatigue strength than that of the monolithic amorphous coating. The enhancement of impact toughness and fatigue properties is owed to the dumping effect of the soft amorphous carbon phase, which alleviated stress concentration and decreased crack propagation driving force.

## Introduction

Fe-based amorphous coatings are considered to be promising materials in applications involving extreme corrosion and wear, such as oil and gas production, shipping, drilling, and earth excavation^1–6^. However, due to the natural brittleness of amorphous phase, the amorphous coatings usually suffered from poor fracture resistance and impact toughness^2, 7^, which severely restricts them for industrious applications. Two Approaches have been proposed to improve the toughness of amorphous coatings: (i) *via* architectural design; and (ii) by the formation of composite structures. One example in the first approach is to form a multilayer structure in coatings, in which amorphous layer and a foreign crystalline layer are alternatively deposited^7^. Our previous work showed that the multilayer structure of Fe-based amorphous coatings with the alternative addition of NiCrAl layers exhibited 10-fold improvement of the impact resistance over the monolithic amorphous coating^7^. However, this approach usually involves complicated process and is hard for up-scale production. In contrast, the second approach *vi*a the formation of composite structures by a simple addition of foreign particles is more applicable in industries. Zhou *et al*.^8^ reported that the Fe-based amorphous coatings with the addition of 8 vol% stainless steel particles leads to 17% improvement of the fracture work over the monolithic amorphous coating, due to the fact that the soft stainless steel particles could effectively absorb the fracture energy. However, the addition of metallic phases brought about a new problem, i.e. reduction of corrosion resistance, as pitting preferentially occurred at the interfaces between the foreign crystalline particles and amorphous matrix in the composite coatings^9^.

To overcome this problem, the addition of non-metallic particles in amorphous coatings could be a good choice, as they are generally not involved with corrosion problem, i.e., no galvanic corrosion occurs between the non-metallic additives and amorphous matrix. In recent years, Fe-based amorphous composite coatings reinforced with various ceramic particles, including WC-Co^10^, TiN^11^, B_4_C^12^ and Al_2_O_3_^13^, have been extensively investigated. Yoon *et al*.^12^ reported that the B_4_C particle-reinforced amorphous coating exhibited better fracture- and wear-resistance; while Yasir *et al*.^13^ revealed that the addition of Al_2_O_3_ particles into Fe-based amorphous coating enhanced impact toughness by triggering the formation of micro-cracks around Al_2_O_3_ particles. In addition to ceramic reinforcements, carbon phases with remarkable mechanical and physical properties are also considered to be a promising candidate of the reinforcements in coatings. A successful example is to introduce carbon nanotubes (CNTs) into Al-Si, Al_2_O_3_ and Cr_3_C_2_ coatings, leading to 50–60% enhancement of the fracture toughness^14–16^. It was revealed that the CNTs took the function of multiple toughening mechanisms, including crack deflection, crack bridging and carbon pullout. In general, the effectiveness of the toughening effect strongly relies on the homogeneity of the CNTs in the composite coatings, wherein carbon (or CNTs) was *in-situ* grown on feedstock powders by high-temperature chemical vapor deposition prior to thermal spraying^16^. However, this approach is not applicable to amorphous coatings, as high-temperature process for the *in-situ* formation of CNTs could damage the amorphous structure and thus invalidate the good properties of amorphous materials.

In this work, we presented a novel approach to fabricate carbon-reinforced amorphous coatings through *in-situ* carbonization of a nylon 11 powder^17, 18^ in the HVOF process. It shows that nylon 11 powders were *in-situ* transformed to amorphous carbon phase, which was homogenously dispersed in the Fe-based amorphous matrix and resulted in enhanced impact toughness and nearly twice-higher fatigue strength. The toughening mechanism was studied in detail in terms of finite element modeling and theoretical analyses.

## Results

**Figure 1:**
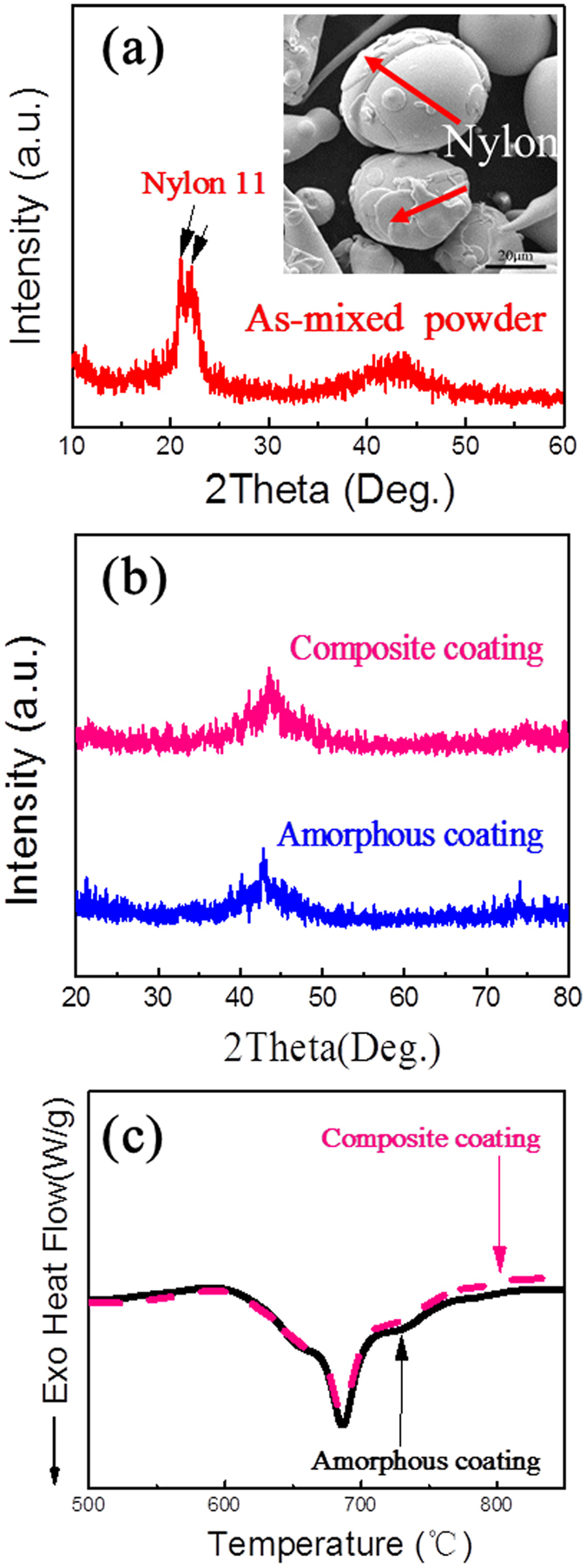
(**a**) XRD pattern of the as-mixed powders (90% amorphous powders and 10% nylon 11 powders). Inset shows the SEM morphology of the as-mixed powders. (**b**) XRD patterns and (**c**) DTA curves of monolithic amorphous coating and composite coating.

Figure 1(a) shows X-ray diffraction (XRD) patterns of the mixture of Fe-based amorphous powders and 10 vol. % nylon 11 particles. The two strong peaks at 2θ = 20.5° and 23.5° correspond to the Bragg reflections of Nylon 11^19^, while the broad hump in a wide range of 40–50° comes from the diffraction of the Fe-based amorphous powders^5^, indicating the original nylon 11 particles are of crystalline structure. The SEM image of the mixing powders (see the inset of Fig. 1(a)) indicates that the nylon powders were tightly adhered to the amorphous powders ensuring the homogenous dispersion of nylon powders in the starting powder feedstock. The XRD patterns of monolithic amorphous coating and composite coating are presented in Fig. 1(b). The nylon crystalline peaks completely disappeared in the composite coating with XRD pattern very similar to that of the monolithic amorphous coating. The result implies that crystalline nylon has been changed probably to amorphous phase after thermal spraying. Figure 1(c) shows the DTA results of the monolithic amorphous coating and the composite coating. Both samples exhibited very similar thermal behaviors with the same crystallization temperature and exothermic reactions, confirming that the two coatings basically have a similar amorphous structure. In order to verify that nylon 11 has been completely decomposed, the composite coating was further examined with infrared spectrum analysis. No any C-H bonds could be detected (not shown here), indicating that nylon 11 has been completely transformed to amorphous carbon during the thermal spray process.

**Figure 2:**
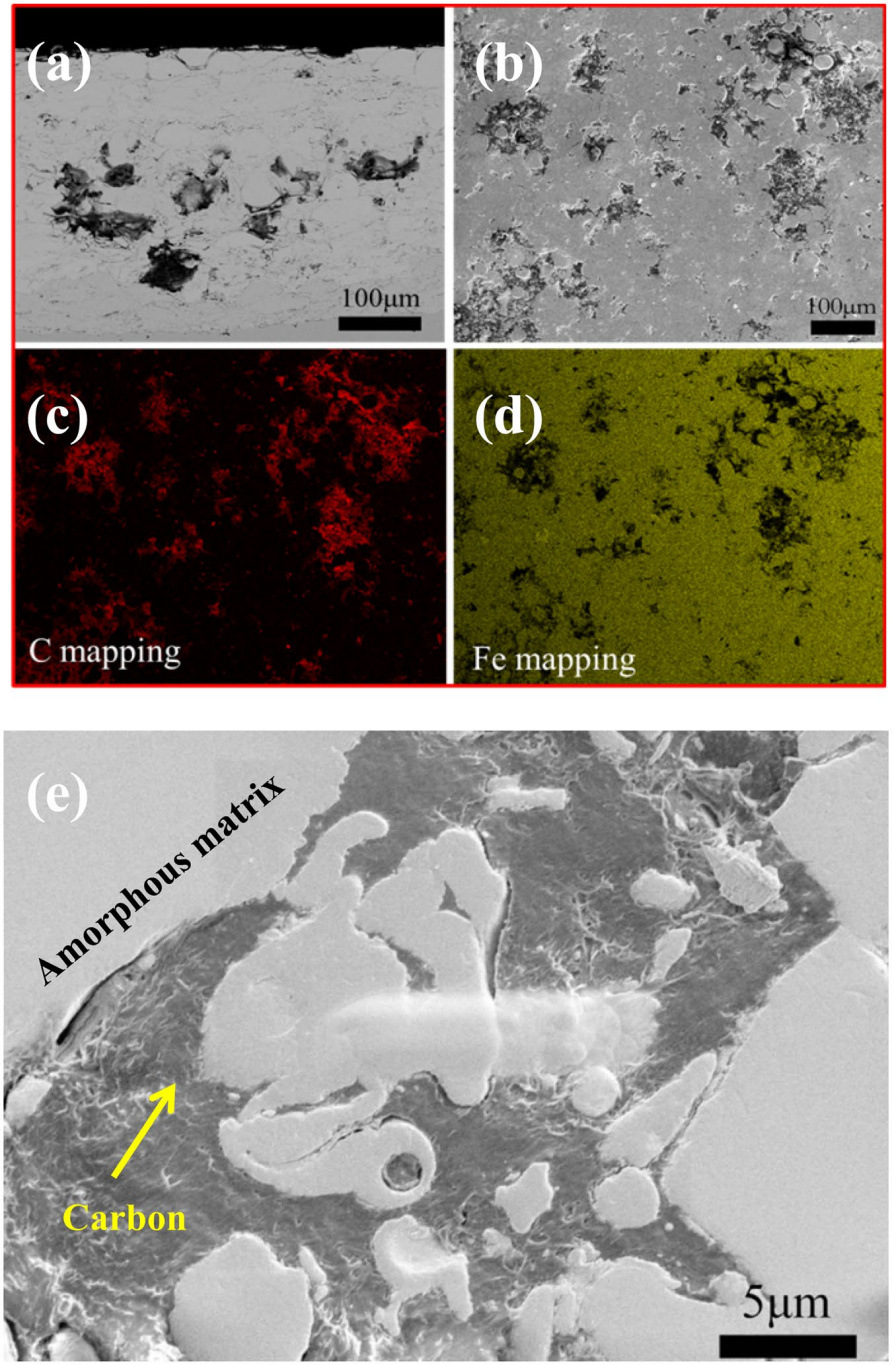
(**a**) Cross-section morphology of the composite coating. (**b**) Surface morphology of the composite coating with the corresponding EDX mapping of C and Fe (**c,d**), showing the uniform distribution of carbon particles in the coating. (**e**) High-magnification image showing the good combination of the amorphous matrix and amorphous carbon phase.

**Figure 3:**
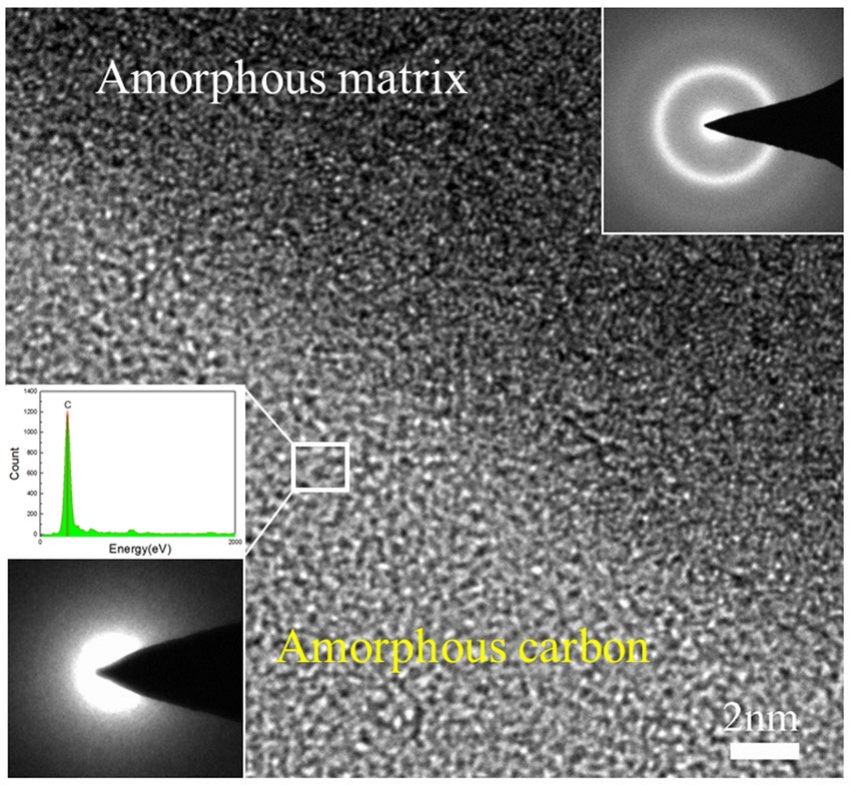
High-resolution TEM image on the interfacial structure between the amorphous matrix and amorphous carbon. Insets are the corresponding SAED patterns from the two phases and an EDX pattern from the amorphous carbon.

Figure 2(a) shows the cross-section SEM morphology of the composite coating with a thickness of around 400 μm, in which two distinct regions could be clearly observed. The white contrast regions with the composition similar to those of amorphous powders are amorphous matrix, while the dark contrast regions enriched with C (>90%) belong to amorphous carbon phase. Figure 2(b) shows the surface SEM morphology with the corresponding EDX mapping (i.e., C and Fe) as shown in Fig. 2(c,d), which indicates that the distribution of amorphous carbon phase was quite uniform in the whole coating. Figure 2(e) displays a magnified image of the interface between amorphous matrix and carbon phase, revealing that the carbon phase was well compacted with amorphous matrix. To clarify the detailed microstructure of the carbon phase and to know how it connected the amorphous matrix in the coating, TEM examination was carried out on the interfacial region which was carefully cut out using FIB. Figure 3 shows a high-resolution TEM image and the corresponding selected area electron diffraction (SAD) patterns. The results demonstrated that carbon and matrix are both of amorphous structure, and they are well connected without visible defects at interface.

**Figure 4:**
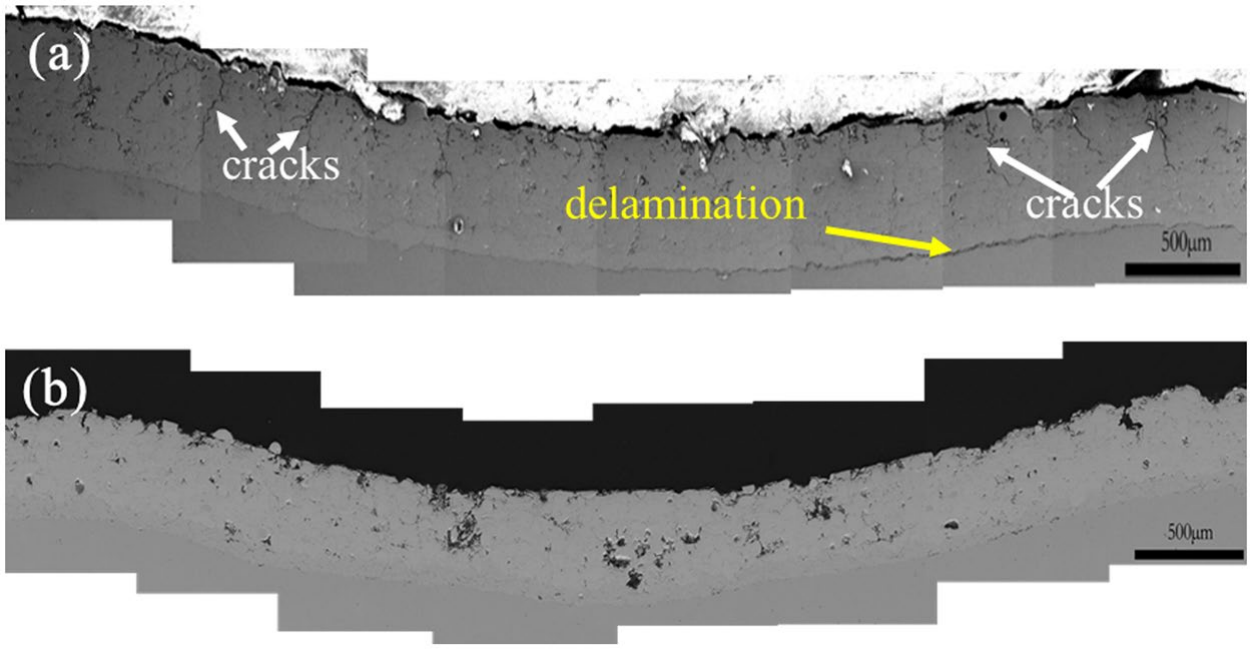
Cross-sectional morphologies of the coatings after impact test under impact energy of ~21 J. (**a**) The amorphous coating and (**b**) the composite coating reinforced by amorphous carbon.

Microhardness measurements were further carried out on the cross-sectional surface of the two coatings, which yield a hardness of 690 ± 31 HV_0.3_ for the composite coating and 800 ± 74 HV_0.3_ for the monolithic amorphous coating, indicating that the amorphous carbon is relatively soft as compared to the Fe-based amorphous matrix. The impact resistance of the composite coating was evaluated by drop-weight impact test with impact energy of 21.2 J. For comparison, the monolithic amorphous coating was also tested in the same condition. Figure 4 shows cross-sectional images of the two coatings after impacting. It can be seen that a few of micro-cracks were formed in the amorphous coating, as indicated by the white arrows (see Fig. 4a), while no visible damages could be observed in the composite coating (see Fig. 4b). This suggests that the soft amorphous carbon can effectively absorb the impact energy, and enhance the impact resistance of the composite coating.

**Figure 5:**
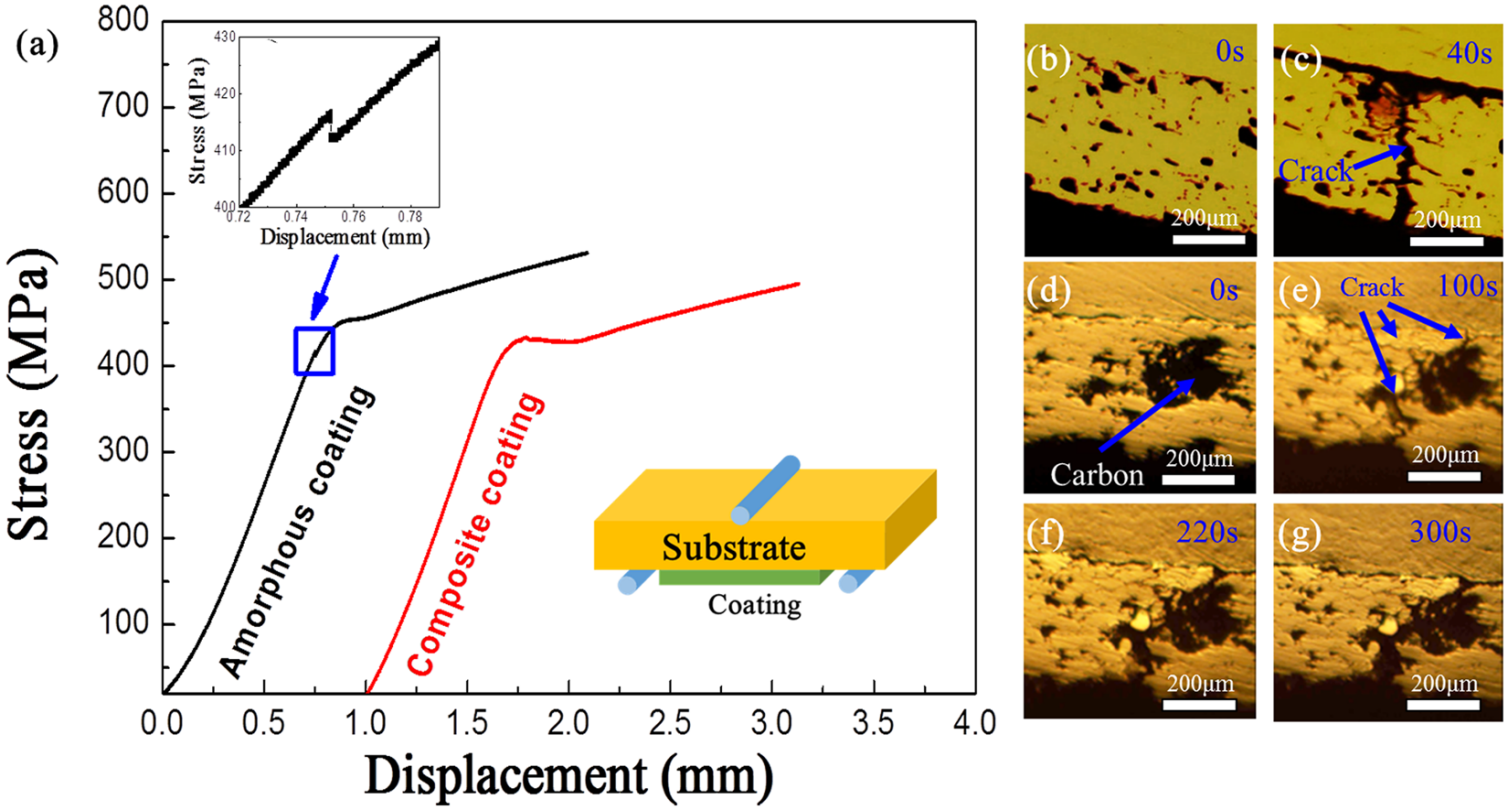
(**a**) Stress-displacement curves obtained from three-point bending tests for the amorphous coating and the composite coating. Insets show a schematic diagram for this test and an enlarged region for amorphous coating showing the fracture of the coating. (**b–g**) Real-time optical photographs obtained during static three-point bending test, showing the crack behaviors in (**b,c**) the amorphous coating and (**d–g**) the composite coating at different loading duration.

The amorphous coating and the composite coating with substrate were further tested by three-point bending and the stress-displacement curves are shown in Fig. 5(a). It can be seen that fracture of the amorphous coating occurred before yielding of the substrate with a sudden drop-down of the stress as shown in the inset (Fig. 5(a)), representing a brittle nature of the amorphous structure. In contrast, fracture for the composite coating did not occur until yielding of substrate, and the fracture process was quite sluggish, as indicated by the gradually decreases of the stress. It is concluded that, as compared to the amorphous coating, the composite coating exhibited not only higher strength (around 5% increment) but also better fracture toughness.

In order to understand the toughening mechanism of the composite coating, the real-time recording on crack propagation under three-bending test were performed by an attached optical microscope and the results are presented in Fig. 5(b–g). For amorphous coating, cracks were found to initiate at pores and then propagate rapidly until complete failure of the whole coating (see Fig. 5c). However, for the composite coating, cracks mostly initiated around amorphous carbon phase, and propagate gradually across the carbon phase (see the propagation process in Fig. 5 (d–g)), demonstrating that the crack propagation is largely hindered by the carbon phase. For example, the composite coating did not fracture before 220 s, which is much longer than the lasting time (<40 s) before fracture in the monolithic amorphous coating.

**Figure 6:**
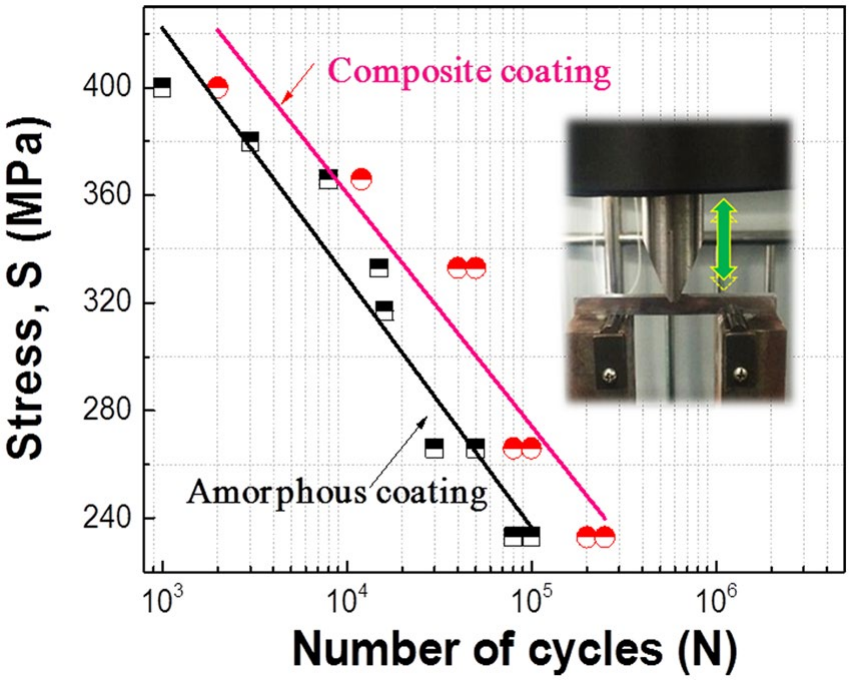
S-N curves for the amorphous coating and the composite coating. Inset shows a picture for fatigue experimental setup.

The fatigue properties of composite coating (with substrate) was also measured in a mode of dynamic three-bending test and compared with the monolithic amorphous coating. (The inset of Fig. 6 displays a real picture of the setup). As substrate was involved in the test, the determination of fatigue lifetime, i.e. the cycles to fracture for coating itself could be different from the bulk sample in standard test. Herein, we proposed a new and fast approach to determinate the fatigue lifetime of the coating at each stress level (see the details in the supplementary materials). Figure 6 shows the *S-N* curves of the two coatings. Considering that the fatigue tests only lasted up to 2 × 10^5^ cycles, only the linear part of the *S*-*N* plots were obtained. Nevertheless, the results revealed clearly that the composite coating exhibited a longer fatigue lifetime at each stress level with respect to the monolithic amorphous coating. To predict the fatigue limit of the two coatings, we fit linearly the data in *S*-*N* curves and obtain an equation for each of the coatings, i.e. *σ*_*r*_ = 700-93log *N* for the monolithic amorphous coating and *σ*_*r*_ = 707-87log *N* for the composite coating, respectively. By prolonging the fitting lines up to *N* = 10^7^, we obtained *σ*_*r*_  = 52 MPa for the amorphous coating and *σ*_*r*_ = 101 MPa for the composite coating, respectively. The fatigue strength of the composite coating is almost twice that of the monolithic amorphous coating.

**Figure 7:**
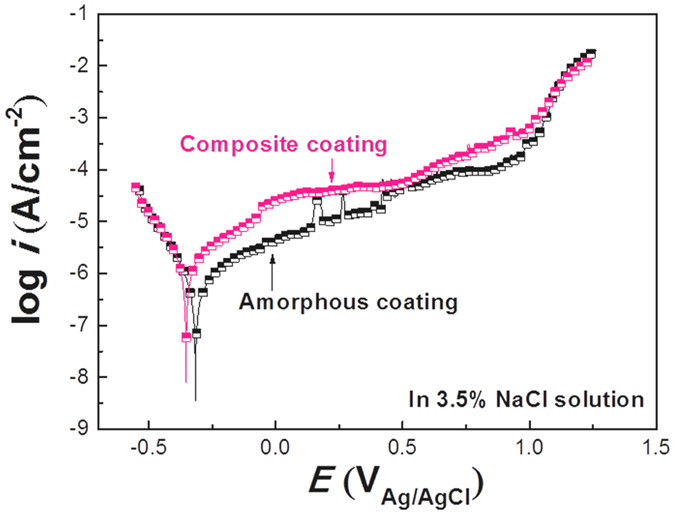
Potentiodynamic polarization curves for the amorphous coating and composite coating in a 3.5% NaCl solution, with a scanning rate of 0.5 mV/s.

To clarify if the carbon phase does not deteriorate the corrosion resistance of the composite coating, an electrochemical polarization test of the composite coating in 3.5 wt% NaCl solution was conducted and compared to the monolithic amorphous coating in the same condition. Figure 7 shows the potentiodynamic polarization curves for the two coatings. Clearly, both coatings show an excellent corrosion resistance with a wide passivation region of more than 1000 mV and a low passivation current density on the order of 10^−5^–10^−4^ A cm^−2^. The similar electrochemical properties indicate that the corrosion resistance for the composite coating was not degraded due to the non-conductivity of the reinforced amorphous carbon, thus any galvanic corrosion or interfacial dissolution can be avoided.

## Discussion

**Figure 8:**
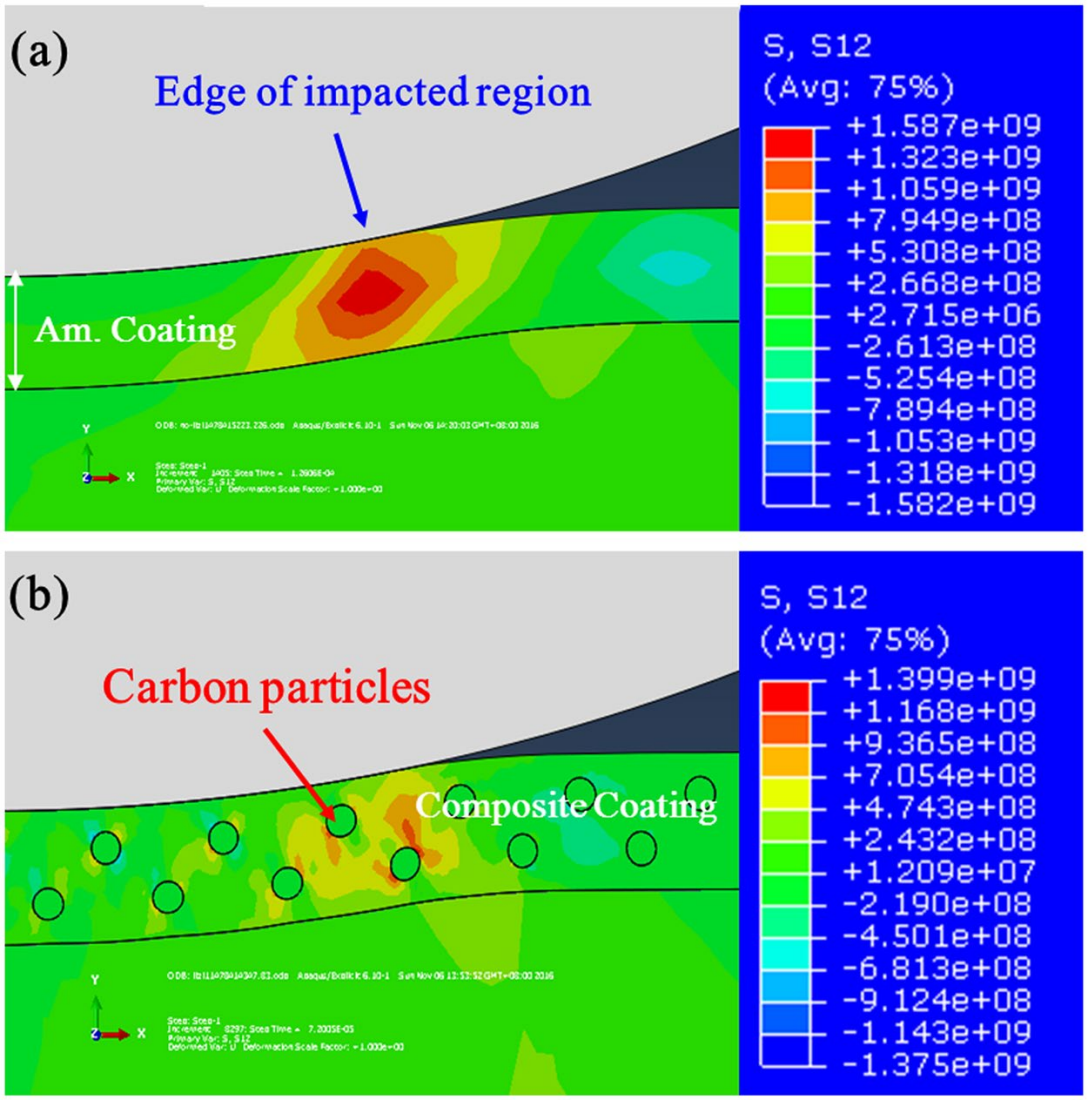
Contour plots of shear stress (S12) in (**a**) the amorphous coatings and (**b**) composite coatings reinforced with carbon particles at the maximum displacement of impact tests.

The above experimental results show that the reinforcement of the amorphous carbon phase could effectively improve the impact resistance and fatigue properties of the amorphous coating. The question arises as to what is the toughening mechanism for the composite coating reinforced with amorphous carbon phase. Regarding the enhancement of impact resistance (normally judged by the visible delamination of the coatings from substrate), the first consideration is that the soft carbon phase could change the stress field distribution and alleviate stress concentration in the coating during impact. To clarify this issue, finite element modeling (FEM) was carried out. Figure 8 illustrates the contour plots of shear stress (S12) in the two coatings (with and without carbon phase) at the maximum displacement during the impact (21.2 J). For the amorphous coating, a significant stress concentration is generated at the edge of the impacted region (see Fig. 8(a)), which is believed to be responsible for the formation of penetrating cracks and interfacial delamination in the monolithic amorphous coating, as observed in Fig. 4(a). In contrast, the stress concentration is effectively mitigated in the composite coating due to the shieling effect of the carbon particles. However, due to the distinct difference in yield strength and deformation ability of the matrix and carbon particles, local stress concentration with very low amplitude could be still created around the carbon phase (Fig. 8(b)), but these weak stresses were not sufficient to cause the formation of the penetrating cracks.

**Figure 9:**
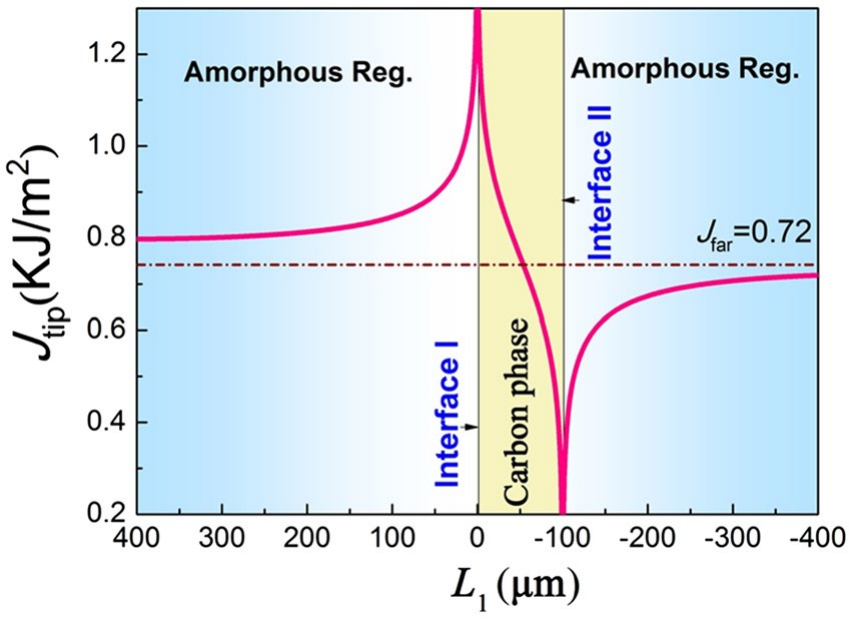
The crack driving force (*J*_tip_) as a function of distance (*L*_1_) against the first interface between the carbon phase and amorphous matrix.

From the standpoint of fracture mechanics, it has been documented that a soft interlayer in brittle matrix could effectively reduce the driving force for crack propagation, and could become the crack arrester. A general criterion for crack growth is that the driving force in the tip of a crack (*J*_tip_) exceeds the intrinsic fracture resistance of the material^20^. According to the theory of Sistaninia and Kolednik, the driving force for crack propagation at a crack tip (*J*_tip_) can be expressed as:^21^

1 $${J}_{{\rm{tip}}}={J}_{{\rm{far}}}+{C}_{{\rm{y}}}$$

where the *J*_fa*r*_ is the far-field J-integral in the matrix and *C*_y_ is the yield stress gradient term. Taking the current composite system as the case where an elastic–plastic phase is embedded in an elastic matrix, we have the following equation that correlates *J*_far_ and *C*_y_:

2 $$\frac{{C}_{y}}{{J}_{far}}=\frac{1}{2\pi }\{Re({\tanh }^{-1}\sqrt{1-{(\frac{{L}_{1}}{{r}_{y}^{IL}})}^{2}})-Re({\tanh }^{-1}\sqrt{1-{(\frac{{L}_{1}-t}{{r}_{y}^{IL}})}^{2}})\}$$

where *L*_1_ is the distance from crack tip to the first interface of carbon/matrix (e.g., the Interface I in Fig. 9), *t* is the thickness of the soft layer, and ${r}_{y}^{IL}$ is the radius of plastic zone that can be determined by Irwin’s model^22^. Based on equations (1) and (2), the driving force (*J*_tip_) crossing the amorphous matrix/carbon interfaces can be theoretically calculated. For simplicity, we only considered a sandwich structure where a thin soft carbon layer with a thickness of 100 μm being placed between two amorphous layers (see Fig. 9) (locally, this is a true case in the amorphous composite coating). Herein, *J*_far_ = 0.72 kJ/m^2^ for the amorphous matrix, the modulus and yield strength of amorphous carbon are about 15 GPa and 90 MPa, respectively^23, 24^. Using these data, the correlation between *J*_tip_ and *L*_1_ can be established, as shown in Fig. 9. One can notice that once a crack propagates from the amorphous matrix (left side in Fig. 9) to amorphous carbon phase, *J*_tip_ increases sharply when crack is approaching the interface I, indicating that crack is easy to penetrate the interface and enter the soft carbon phase, and consequently causes a rapid reduction of *J*_tip_ to a level far below *J*_far_ (see Fig. 9). If *J*_tip_ is sufficient low, the crack will be completely arrested by the carbon phase, i.e., the stop of crack propagation. *J*_tip_ reaches the lowest level at the interface II^25^. The driving force could increase in the amorphous matrix crossing the interface II at right side with increasing external force, and the crack will further propagate if the driving force (*J*_tip_) is higher than intrinsic fracture resistance.

**Figure 10:**
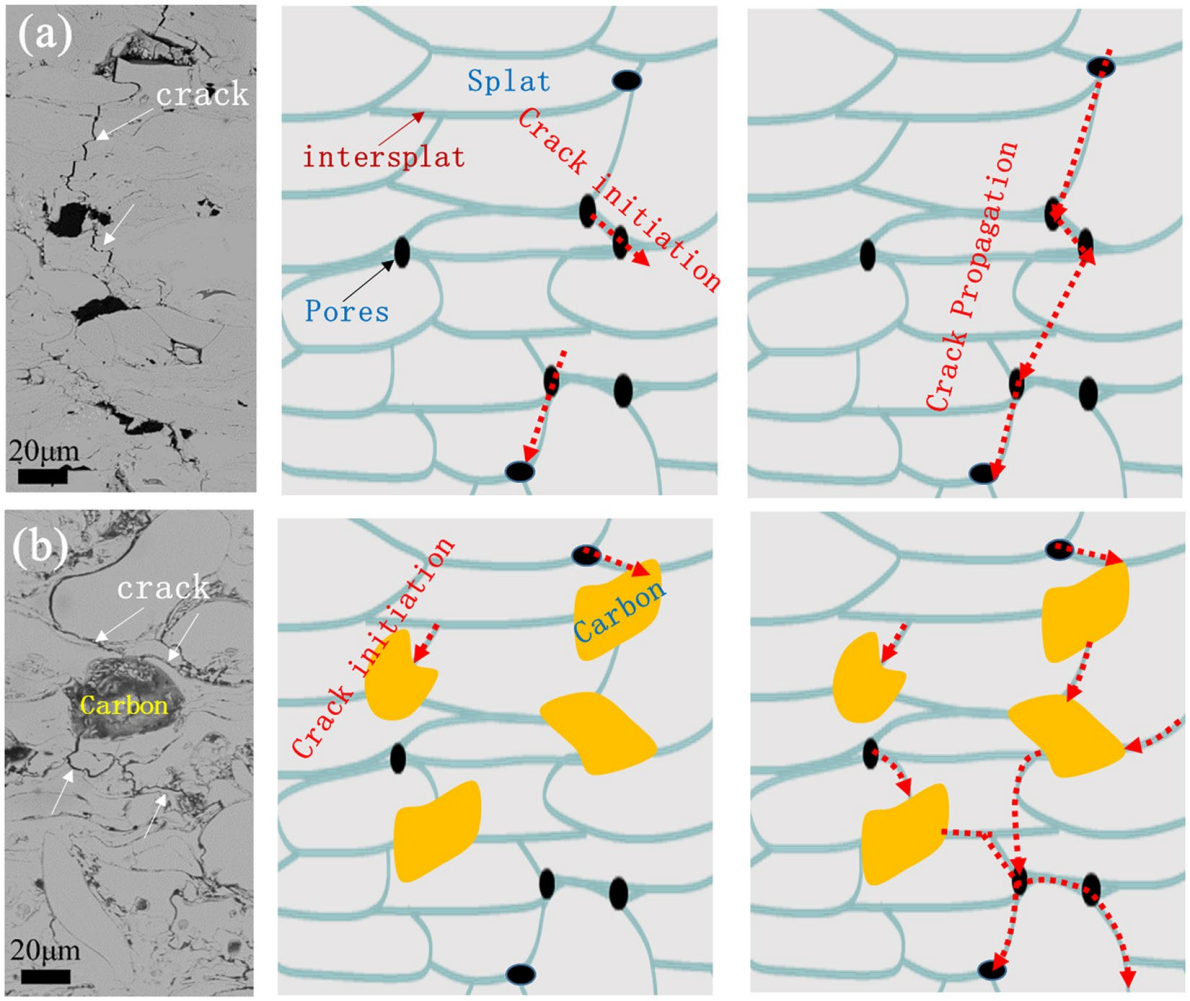
SEM fracturing morphologies after fatigue tests and schematic diagrams depicting the cracking behaviors (**a**) the amorphous coating and (**b**) the composite coating with carbon. In amorphous coating, the crack propagates through the pores or defects and easily penetrates the whole coating. But in composite coating, the crack propagation could be hindered by ductile carbon particles, which is therefore difficult for developing through-wall crack in the whole coating.

Regarding the enhancement of fatigue properties of the amorphous composite coating (the fatigue strength for composite coating is almost two times higher than the monolithic amorphous coating). The mechanism could be understood by investigating the crack propagation behaviors under cyclic loading. Figure 10(a) and (b) show the tracks of crack propagation for the amorphous coating and the composite coating, respectively. For the amorphous coating, the crack propagates along the pores and finally penetrate the whole coating (see Fig. 10(a)); while for the composite coating, crack propagation was frequently stopped when encountering amorphous carbon phase (see Fig. 10(b)). A schematic showing the difference in crack initiation and propagation in the two coatings under cyclic loading is illustrated in Fig. 10(a) and (b). For the monolithic amorphous coating, crack mainly initiates at defects, such as pores or intersplats, and propagates through the intersplats or *via* the connection of pores until failure of the coating. In contrast, for the composite coating, when the crack encountered ductile carbon phase, crack propagation stops. New cracks will initiate somewhere around the carbon phase with continuously cyclic loading. These newly formed cracks then propagate mainly along the intersplats until penetrating the whole coating or stopping again when meeting another carbon phase (Fig. 10b). Therefore, the crack propagation rate in composite coating is much slower than that in monolithic coating. Therefore, the fatigue strength in the composite coating could be significantly enhanced (around twice).

## Conclusions

In this work, a Fe-based amorphous composite coating reinforced with amorphous carbon phase was developed *via in-situ* carbonization of nylon 11 powders during HVOF thermal spraying. The amorphous composite coating exhibited high bending strength (~410 MPa) and excellent corrosion resistance. The composite coating does not show any damage upon impacting with the energy of ~21 J and have twice higher fatigue strength than that of the monolithic amorphous coating. Finite element simulation revealed that the ductile amorphous carbon phase could effectively alleviate stress concentration during impact process. Further, theoretical analysis suggested that the amorphous carbon phase could significantly reduce the driving force for crack propagation due to the dumping effect of the soft amorphous carbon phase, which suppressed crack propagation, and consequently enhanced the impact resistance and fatigue strength of the amorphous composite coating.

## Experimental Methods

Fe_48_Mo_14_Cr_15_Y_2_C_15_B_6_ (at. %) amorphous powders with a size range of 33–55 μm prepared by inert gas atomization were used as the starting powder feedstock^13^. The amorphous powders were then blended with 10 vol. % of nylon-11 powders (size: 75–100 μm) by low-energy ball milling. The blended powders were sequentially thermally sprayed onto AISI 1045 mild-steel substrate by a KY-HVOF thermal spraying system (made in China). The principle of HVOF is that the kerosene and oxygen with a desired mass ratio combust to create a high temperature and high pressure exhaust gas, which then passes through a narrow nozzle to obtain a high speed of gas flow. In front of the nozzle, powders are introduced radially, thereby experiencing a high acceleration to supersonic velocities; upon impacting the substrate, the powders spread out thinly to form a well-bonded dense coating. This technique is more suitable for the preparation of amorphous metallic coatings compared to other thermal spraying technique such as high-temperature plasma spraying, because its high kinetic energy could provide an ultrahigh cooling rate for glass formation while the relatively low processing temperature could avoid severe oxidation and composition segregation of the in-flight particles^26^. The parameters used are as follows: kerosene and oxygen flow rate are 24 l/h and 32 l/h, respectively; the spraying distance is 350 mm and the spacing is 5 mm; the powder feed is 30 g/min^27^. The microstructures of the coatings were characterized by X-ray diffraction (XRD, χ’ Pert PRO) using Cu Kα radiation, differential thermal analysis (DTA, TA Q600SDT), and scanning electron microscopy (SEM, Quanta 2000) coupled with energy dispersive X-ray spectroscopy (EDX). The specimens for transmission electron microscopy (TEM, Tecnai G2 F30) examination were fabricated by focused ion beam (FIB, Quanta 3D FEG).

Hardness of the coatings was measured using a micro-indenter with the load of 300 g and a dwell time of 10 s on as-polished cross-sectional surface of the coatings. At least 10 measurements were repeated for each sample to ensure the data repeatability. The toughness of the coatings was evaluated by impact test, the merit of this technique over others (such as nanoindentation) is that the average toughness of the whole sample can be evaluated. Impact resistance of the coatings was measured on a home-made drop-weight impact tester, wherein a steel tup with a hemispherical head (16 mm diameter) and an overall weight of 1.8 kg was dropped down from 120 cm height (corresponding to 21.2 J impact energy) onto the as-sprayed coatings^13^. The stress distribution in the coatings upon impact was simulated by finite element method (FEM) with the ABAQUS software. A two dimensional axis symmetric model with the z-axis coincident to loading direction was constructed. The model consists of monolithic amorphous coating and the composite coating reinforced with carbon particles in the same thickness of 400 μm. The samples are assumed to be loaded by a steel ball with a velocity of 1.5 m s^−1^ and to be fixed in the bottom, just imitating the real condition. The interface between coating and substrate is regarded as prefect bonding without delamination. The amorphous carbon phase is regarding as spheres with an average radius of ~100 μm and homogeneously distributed in the amorphous matrix with prefect bonding.

Three-point bending test on rectangular coating specimens with substrate (100 mm × 7 mm × 9 mm) was carried out by a universal mechanical testing machine (Reger M4050). The outer span is 70 mm and the loading rate is 0.1 mm/min. In addition, a traveling microscopy was employed to get a real-time recording of crack propagation in the coatings. Fatigue test was carried out using a computer-controlled mechanical testing machine with a load ratio of *R* = 0.1 (where *R* = *P*_min_/*P*_max_, *P*_min_ and *P*_max_ are the minimum load and the maximum load, respectively, in a loading cycle) at a nominal frequency of 10 Hz. The samples for fatigue test have exactly the same dimension as those used for bending test. After fatigue test, the side surface of the samples was examined by SEM to observe crack propagation behaviors.

The corrosion resistance of the coatings was evaluated by potentiodynamic polarizations in a 3.5% NaCl solution at room temperature, which were performed in a standard three-electrode cell with platinum the counter electrode and an Ag/AgCl reference electrode.

## Electronic supplementary material

Supplementary materials
