# Supplementary material for: Toughening Fe-based Amorphous Coatings by Reinforcement of Amorphous Carbon
Source: Sci Rep. 2017 Jun 22;7:4084. doi: 10.1038/s41598-017-04504-z (PMC5481419; doi:10.1038/s41598-017-04504-z)
Supplement: Supplementary file 1 — Supplementary materials [file 41598_2017_4504_MOESM1_ESM.docx]

**Online Supplementary materials for “****Toughening Fe-based Amorphous Coatings by Reinforcement of Amorphous Carbon” submitted to Scientific reports**

**Wei Wang, Cheng Zhang*, Zhiwei Zhang, Yicheng Li, Muhammad Yasir, Haitao Wang, Lin Liu***

*School of Materials Science and Engineering and State Key Lab for Materials Processing and Die & Mould Technology, Huazhong University of Science and Technology, Wuhan 430074, China*

* Corresponding authors. (CZ) [czhang@hust.edu.cn](mailto:czhang@hust.edu.cn) and (LL) [lliu2000@mail.hust.edu.cn](mailto:lliu2000@mail.hust.edu.cn)

**Criterion for fatigue life of the coatings:**

To determine fatigue life for thermally sprayed amorphous coatings on substrate independently, a new criterion has been proposed on the basis of the maximum and minimum stroke obtained during the cyclic loading. The principle is illustrated in **Fig. S1.** Herein, the stroke is defined as the displacement deviated from the original position S due to the deformation of the whole specimen upon external load (see Fig. S1 (a)). Because a sinusoidal form of the load was adopted, there should be a maximum stroke (max-stroke) at the maximum load and minimum stroke (min-stroke) at minimum load in a cycle. The max-stroke and min-stroke as a function of cycling numbers (*N*) for the coatings (with substrate) were individually recorded by computer, and the results are shown in Fig. S1 (b). Three distinct regions can be observed in the two plots: Stage 1) Remaining constant of the max/min-strokes in the first 20000 cycles; Stage 2) Decrease of the max/min-strokes (noted that the stroke is presented in a negative value); and Stage 3) Increase again of the max/min-strokes at the end (*N*>100000). It is believed that the sudden change in the max/min-strokes reflects the change in the stress state, which is associated with the damage of the tested coatings, i.e., either crack initiation in the coating or delamination of the coating from the substrate. For example, when a crack is initiated in the coating, part of the external load was used as the driving force for crack propagation, which leads to a decrease of the stress. As a result, the max/min strokes could suddenly decrease. However, once the crack penetrates the whole coating, the max/min strokes could increase due to the larger deformation of the substrate with a low strength (made of low carbon steel). Therefore, the threshold between the stage 2 and stage 3 can be regarded as the fatigue lifetime under a certain condition (as arrowed in Fig. S1 (b)). The above argument was further verified via *in-situ* optical observations on the cross-sectional fracture morphology of a coating, as shown in Fig. S1 (c-e). At stage 1, no any crack was detected (Fig. S1 (c)). At stage 2, some micro-cracks were initiated around the pores. At stage 3, the crack penetrated the whole coating, indicative of the failure of the coating. This criterion can be used to determine the fatigue lifetime of the monolithic amorphous coating and the amorphous composite coating.


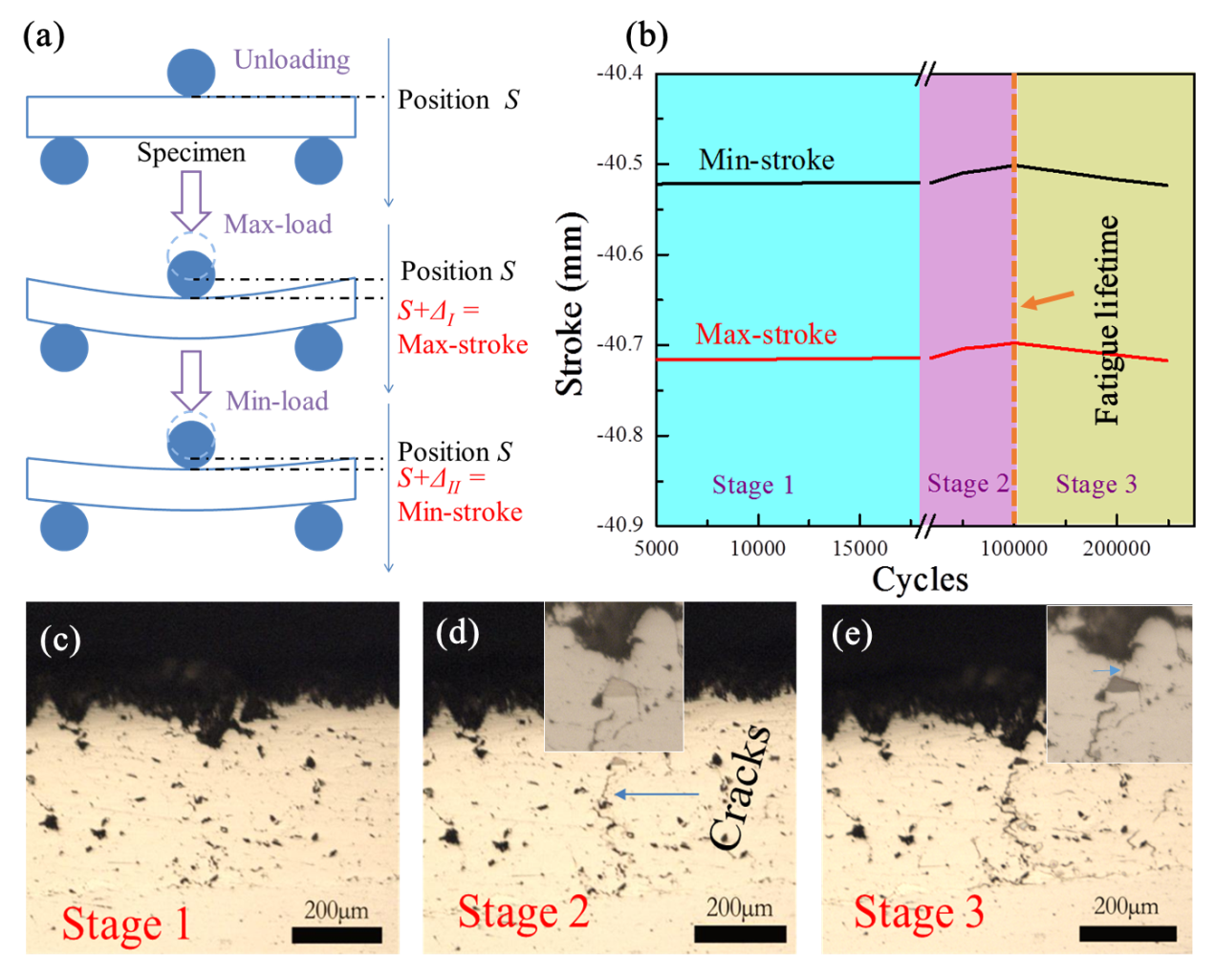


**Fig. S1: (a) A schematic diagram showing the definition of max-stroke and min-strake in one cycle. (b) The max-stroke and min-stroke as a function of cycling numbers for the coating (together with substrate) as recorded directly by computer. (c-e) *In-situ* optical observations on crack propagation in the amorphous coating at different stages. Insets in (d, e) are the enlargement of the region near the top surface, in which crack does not penetrate the coating at stage 2, while penetrate the whole coating at stage 3.**
